# Modeling Blood–Brain Barrier Efflux Transport Using a Breast Cancer Resistance Protein Overexpression Cell Line

**DOI:** 10.3390/biomedicines14061192

**Published:** 2026-05-25

**Authors:** Alexandra E. Meyer, Natalie G. Alexander, Elisa M. Tucker, Hallie E. Knight, Benjamin T. Klemp, Bryan J. Estrada, Sarah F. Hathcock, Henry D. Mauser, Kylie A. Buchanan, Brandon J. Kim

**Affiliations:** 1 Department of Biological Sciences, University of Alabama, Tuscaloosa, AL 35401, USA; 2 Department of Biological Sciences, University of Texas at Dallas, Richardson, TX 75080, USA

**Keywords:** blood–brain barrier, breast cancer resistance protein, drug delivery, efflux transport, *ABCG2*

## Abstract

**Background**: The blood–brain barrier (BBB) separates the circulation from the central nervous system (CNS) and serves to maintain brain homeostasis. The BBB comprises highly specialized brain endothelial cells (BECs) with unique properties that allow the BBB to maintain strict regulation of molecules entering and exiting the CNS. These characteristics include tight junctions, low endocytosis rates, and efflux and nutrient transporters. Breast cancer resistance protein (BCRP) is an efflux transporter found at the BBB that plays a key role in protecting the CNS. Together with other efflux transporters, BCRP contributes to multidrug-resistant cancers and difficulty delivering drugs and therapeutics to the brain and other organs. **Methods**: Using the hCMEC/D3 line, we utilized BCRP substrate rosuvastatin to effectively select for cells expressing high amounts of BCRP, thus generating hCMEC/D3-BCRP. To assess protein abundance, we utilized flow cytometry and confirmed expression via qPCR. To investigate BCRP efflux function in evolved hCMEC/D3-BCRP, we performed substrate accumulation assays with BCRP and P-gp substrates. **Results**: We found hCMEC/D3-BCRP had increased BCRP abundance and expression relative to parent hCMEC/D3. We also observed an increase in BCRP function via substrate accumulation of two BCRP substrates compared to parent hCMEC/D3. **Conclusions**: BCRP serves a protective role within the BBB and is a major hurdle in drug delivery. We generated a BCRP overexpression BEC cell line (hCMEC/D3-BCRP) under the influence of endogenous promoters. This cell line can be used to further investigate the role of BCRP in BECs and utilized in efflux transport studies.

## 1. Introduction

The blood–brain barrier (BBB) is a highly specialized and dynamic interface formed primarily by brain endothelial cells (BECs) that tightly regulate molecular exchange between the central nervous system (CNS) and the systemic circulation [1,2,3]. Unlike peripheral endothelial cells, BECs exhibit a distinct phenotype characterized by complex tight junction networks, an absence of fenestrations, and reduced rates of vesicle-mediated transport, all of which collectively restrict paracellular and transcellular permeability. These structural and functional adaptations are essential for maintaining CNS homeostasis, protecting neural tissue from blood-borne toxins, pathogens, and immune mediators, and preserving proper neuronal signaling [1,2,3]. In addition to the physical barrier properties, BECs express polarized transport systems that actively regulate solute flux across the BBB. Among these, ATP-Binding Cassette (ABC) transporters serve as energy-dependent efflux pumps that actively prevent the entrance of toxins, metabolites, and other molecules back into the circulation [4]. This efflux capacity is a critical hallmark of the BBB’s neuroprotective function but also represents a significant obstacle to effective drug delivery to the CNS.

Breast cancer resistance protein (BCRP, *ABCG2*) is an ABC efflux transporter localized to the luminal membrane of BECs and plays a key role in limiting CNS exposure to structurally different substrates, including anticancer agents, antibiotics, and statins [4,5,6,7]. BCRP utilizes ATP hydrolysis to actively transport substrates against concentration gradients. While this activity is essential for protecting the brain from potentially harmful compounds, BCRP also restricts the brain penetration of many candidate therapeutics, contributing to therapeutic failure in CNS disorders such as epilepsy, brain tumors, and neurodegenerative diseases [4,5,7,8,9].

The functional presence of BCRP at the BBB has been demonstrated using a variety of experimental approaches. Ex vivo luminal transport assays in isolated rat and mouse brain capillaries have demonstrated active BCRP-mediated efflux [10]. In vitro studies using immortalized brain endothelial cell lines and multidrug-resistant models have further established the role of BCRP in restricting CNS drug accumulation and have enabled interrogation of transporter regulation and substrate specificity [11,12,13,14,15].

The human cerebral microvascular endothelial cell line hCMEC/D3 is widely used as an in vitro BBB model due to its stable endothelial phenotype and expression of key tight junction proteins and transporters found in human brain microvessels [16,17]. However, native efflux transporter expression levels in the hCMEC/D3 cell line have limitations for studying high-capacity efflux processes or screening transporter-targeted therapeutics. Recently, we demonstrated the generation of an endogenous P-glycoprotein (P-gp, *ABCB1*) overexpression BEC line under native promoter control, providing a platform for evaluating transporter inhibition and drug transport in brain-derived endothelial cells [18]. While our model captures key endothelial features, it does not fully recapitulate the transcriptional heterogeneity observed across vascular subtypes in vivo.

Here, we utilized the well-characterized BCRP substrate rosuvastatin to selectively enrich for hCMEC/D3 cells expressing elevated levels of BCRP. This selection strategy yielded the hCMEC/D3-BCRP cell line, which exhibits robust and functional BCRP-mediated efflux activity. The hCMEC/D3-BCRP model is designed as a complementary tool to existing BBB models and provides a valuable BBB human-based system for investigating BCRP-dependent transport mechanisms, evaluating drug–transporter interactions, and advancing strategies to overcome efflux-mediated limitations in CNS drug delivery.

## 2. Materials and Methods

### 2.1. Generation and Maintenance of BCRP Overexpressing hCMEC/D3-BCRP Cells

hCMEC/D3 cells were maintained in EndoGRO MV (Sigma-Aldrich, St. Louis, MO, USA, SCME004) as previously described [18,19]. At approximately 80% confluency, cells were passaged onto flasks coated with 1% rat tail collagen (RTC). Generation of hCMEC/D3-BCRP cells was performed according to previously established protocols [18]. Parental hCMEC/D3 cells were passaged in EndoGRO medium supplemented with 50 µM or 100 µM rosuvastatin (ThermoFisher, Waltham, MA, USA, AC456232500) for a minimum of 5 passages. hCMEC/D3-BCRP cells were maintained in medium containing rosuvastatin until one day prior to experimentation, at which point the medium was replaced with EndoGRO without rosuvastatin. hCMEC/D3-BCRP cells were tested and are negative for the presence of mycoplasma.

### 2.2. Efflux Transporter Functional Assays

BCRP activity in BECs was measured using functional assays based on previously established protocols [15]. Accumulation of fluorescent BCRP substrates Hoechst 33342 (ThermoFisher, Waltham, MA, USA, 62249) and Pheophorbide A (PhA) (MedChemExpress, Monmouth Junction, NJ, USA, HY-125665) was measured in the presence of BCRP-specific inhibitor Ko143 (Enzo, Long Island, NY, USA, 89158-270), which served as a positive control [15,20,21]. Four days post-seeding, cells were washed once with pre-warmed Hanks’ Balanced Salt Solution (HBSS) and pretreated with 1µM Ko143 or vehicle for one hour at 37 °C and 5% CO_2_. Following pretreatment, 10 µM of Hoechst 33342 or Pheophorbide A, with or without Ko143, or vehicle control in HBSS was added for a 2 h incubation. After incubation, each well was washed twice with 500 µL pre-chilled PBS and lysed in 200 µL RIPA buffer. P-gp substrate accumulation was measured using Rhodamine 123 (R123) (Sigma-Aldrich, St. Louis, MO, USA, 83702) in the presence of P-gp inhibitor cyclosporine A (CsA) (Sigma-Aldrich, St. Louis, MO, USA, C1832) at 10 µM. Fluorescence for substrate accumulation assays was measured using a microplate reader (Molecular Devices, San Jose, CA, USA, SpectramaxiD3 and ThermoFisher, Waltham, MA, USA, Varioskan LUXline). Bicinchoninic acid (BCA, [ThermoFisher, Waltham, MA, USA, 23227]) assays were performed to normalize fluorescence values to total protein concentration per well.

### 2.3. RNA Isolation and Quantitative PCR

RNA was isolated using the NucleoSpin RNA kit (Macherey-Nagel, Düren, Germany, MANA740955.250). Complementary DNA (cDNA) was synthesized using the qScript cDNA Synthesis Kit (Quantabio, Beverly, MA, USA, 95047-500). SYBR Green quantitative PCR (qPCR) was performed using cDNA and human *ABCG2* primers: forward, 5′-TATAGCTCAGATCATTGTCACAGTC-3′ and reverse, 5′-GTTGGTCGTCAGGAAGAAGAG-3′ [22]. qPCR analysis of *ABCC1*, *ABCC2*, *ABCC3*, and *ABCC4* was determined using Taqman probes (ThermoFisher, Waltham, MA, USA, and Hs02786624_g1, Hs00988721_m1, Hs00978452_m1, Hs01561483_m1, Hs00960489_m1) and Taqman Mastermix (ThermoFisher, Waltham, MA, USA, 4444557). Gene expression levels were normalized to 18S or GAPDH, which were used as a housekeeping gene as previously described [15]. Data are presented as fold change relative to parental hCMEC/D3 expression.

### 2.4. Flow Cytometry

Flow cytometry was performed as previously described [18]. At 90–100% confluence, hCMEC/D3 or hCMEC/D3-BCRP cells were washed with PBS and incubated with Accutase (Stemcell Technologies, Vancouver, BC, Canada, 07920) for 10 min at 37 °C and 5% CO_2_. Cells were then washed, pelleted at 500× *g* for 10 min, and fixed in 1% paraformaldehyde (PFA) in PBS. Two additional washes were performed using wash buffer (5% bovine serum albumin [BSA] and 0.1% Triton-X in PBS), after which cells were counted to estimate cell density. Cells were incubated overnight at 4 °C with either an IgG1 isotype (Bio X Cell, Lebanon, NH, USA, BEoo83) or anti-BCRP antibody (Sigma-Aldrich, St. Louis, MO, USA, MAB4155) at 0.5 µg per million cells. The following day, cells were stained with goat anti-mouse Alexa Fluor 488 (ThermoFisher; A11001) at a 1:5000 dilution. Data were collected using an Attune NxT Flow Cytometer (ThermoFisher, Waltham, MA, USA), and histograms and dot plots were generated using FlowJo software version 10.10.0.

### 2.5. Trans-Endothelial Electrical Resistance (TEER)

Five days post-seeding onto 1% RTC coated 12-well Transwell inserts, TEER was measured in hCMEC/D3 and hCMEC/D3-BCRP cells using an EVOM Manual Voltohmeter (World Precision Instruments, Sarasota, FL, USA, EVM-MT-03-02). TEER measurements were performed in complete culture medium at room temperature following a ten-minute equilibrating period post removal from 37 °C incubation. Measurements were taken in triplicate across three independent passages.

### 2.6. Immunostaining

Both hCMEC/D3 and hCMEC/D3-BCRP cells were immunostained 5 days post-seeding. Cells were fixed for 15 min with either ice-cold methanol [(Occludin; ThermoFisher, Waltham, MA, USA, 33-1500), (ZO-1; ThermoFisher, Waltham, MA, USA, 33-9100)] or 4% PFA [(BCRP, Sigma-Aldrich, St. Louis, MO, USA, MAB4155), (VE-cadherin, Santa Cruz Biotechnology, Dallas, TX, USA, sc-52751)] depending on the antibody. For BCRP, cells were permeabilized with 0.1% Triton-X-100 in PBS for 10 min. Cells were blocked with 3% BSA in PBS and incubated with primary antibodies overnight. Species-matched secondary antibodies were used the following day, and nuclei were stained with DAPI. Visualization was acquired using a Nikon Ti2 inverted epifluorescence microscope equipped with a Qi2 camera (Nikon, Tokyo, Japan) and NiS Elements software version AR.5.30.05. Images were analyzed using ImageJ Software (FIJI) version 2.16.0/1.54s.

### 2.7. Immunoblotting

Protein concentrations for loading were determined using a BCA assay. Equal protein concentrations were loaded in triplicate onto a 12-well Bolt 4–12%, Bis-Tris Plus WedgeWell gel (ThermoFisher, Waltham, MA, USA, NW04122Box) and run at 140 V for 30 min, followed by transfer onto a nitrocellulose membrane at 300 mA for 90 min. Following transfer, membranes were stained with Ponceau S for imaging. Membranes were then blocked in 5% nonfat dry milk for 1 h. Primary antibodies were incubated with the membranes overnight at 4 °C. The following day, blots were rinsed with Tris-buffered saline containing 0.1% Tween 20 (1× TBST) three times in five-minute intervals and then incubated with the appropriate secondary antibody for 1 h. All targets received a goat anti-mouse secondary antibody (ThermoFisher, Waltham, MA, USA, #A-11001). The blots were imaged using the Azure 300 Imaging System with SuperSignal West Pico PLUS Chemiluminescent Substrate (ThermoFisher, Waltham, MA, USA, 34577).

### 2.8. Statistical Analysis

Statistical analysis and figure generation were performed using GraphPad Prism version 10.4.1. For pair-wise comparisons, Student’s *t*-tests were performed to determine significance. Normality of data distribution was assessed prior to parametric testing using the Shapiro–Wilk test. Error bars represent the standard deviation (SD). Experiments were performed in biological and technical triplicate. The ROUT outlier test (Q = 1%) was applied to datasets, and outliers were removed when identified.

## 3. Results

### 3.1. Generation of hCMEC/D3-BCRP from hCMEC/D3 Cell Line

**Figure 1:**
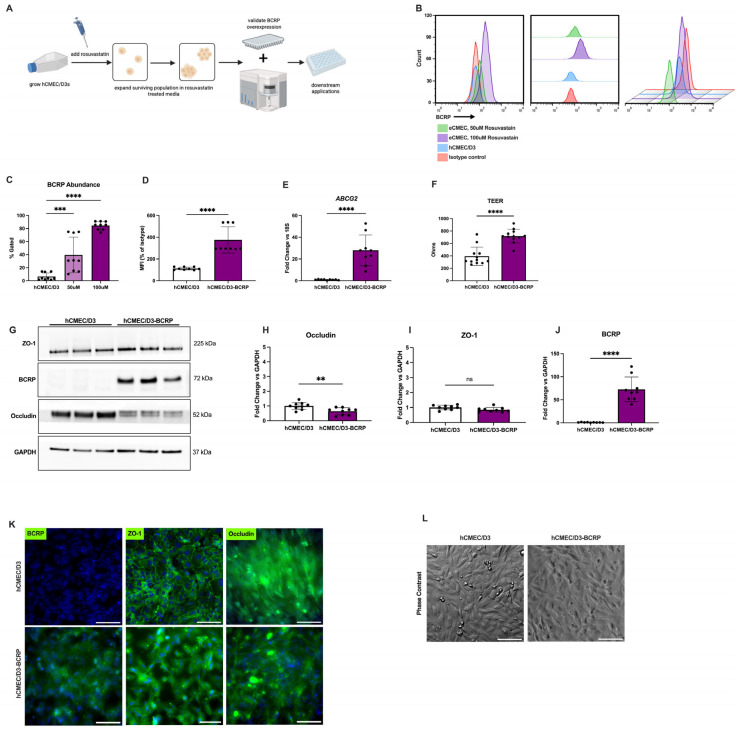
hCMEC/D3-BCRP overexpresses BCRP. (**A**) Schematic of hCMEC/D3-BCRP generation and downstream applications (created with Biorender.com). (**B**) Representative flow cytometry histograms of hCMEC/D3-BCRP populations (green = 50 µM rosuvastatin; purple = 100 µM rosuvastatin) compared to hCMEC/D3 populations (red = isotype; blue = BCRP). (**C**) Flow cytometry results showing increased BCRP abundance in hCMEC/D3-BCRP populations compared with hCMEC/D3 populations. (**D**) Mean fluorescent intensity was significantly increased in hCMEC/D3-BCRP populations compared to hCMEC/D3. (**E**) qPCR quantification of *ABCG2* in hCMEC/D3 and hCMEC/D3-BCRP cells treated with 100 µM rosuvastatin shows upregulation of BCRP transcripts in hCMEC/D3-BCRP. (**F**) hCMEC/D3-BCRP cells exhibited significantly higher TEER measurements than the parental hCMEC/D3 cell line. (**G**) Western blot analysis probing for tight junction proteins Occludin and ZO-1, as well as efflux transporter BCRP, showed an increased abundance of BCRP, a decrease in Occludin abundance, and no significant difference in abundance of ZO-1 in the hCMEC/D3-BCRP line relative to the parental hCMEC/D3 as quantified in (**H**–**J**). GAPDH is shown to show relative protein concentration loaded for each lane. (**K**) Representative fluorescent images of BCRP, ZO-1, and Occludin (green) with nuclei stained with DAPI (blue). (**L**) Phase contrast microscopy of hCMEC/D3 and hCMEC/D3-BCRP cells shows similar morphology when confluent. Scale bar = 100 μm. (**B**–**L**) Experiments were performed across three independent passages in technical triplicate (*n* = 9). Statistical significance was determined by Student’s *t*-test (**D**–**F**,**H**–**J**) or ordinary one-way ANOVA followed by Dunnett’s multiple comparisons test (**C**), ** *p* < 0.01, *** *p* < 0.001, **** *p* < 0.0001, ns, non-significant. Normality tests were performed, and error bars represent SD.

To generate hCMEC/D3-BCRP cells, we adapted previously established protocols for hCMEC/D3-MDR1, a P-gp overexpressing cell line [18,23,24,25]. Cells were selected for BCRP overexpression by supplementing EndoGRO MV medium with rosuvastatin, a cytotoxic BCRP substrate (Figure 1A) [26,27]. Parental hCMEC/D3 cells were passaged into flasks containing 50 µM or 100 µM rosuvastatin, conditions under which cell populations were initially severely decreased. After multiple passages, the selected cell populations recovered and resumed normal proliferation. Rosuvastatin-treated hCMEC/D3 populations exhibited increased BCRP abundance, as measured by flow cytometry (Figure 1B–D and Figure S1). To determine whether BCRP expression was increased at the transcriptional level, qPCR analysis was performed, revealing elevated expression of the transcript for *ABCG2* (Figure 1E). As a barrier property, TEER was measured for three independent passages, where we observed higher TEER in hCMEC/D3-BCRP cells relative to parental hCMEC/D3 (Figure 1F). Western blot analyses were performed to observe tight junctions, Occludin and ZO-1, expression as well as BCRP abundance (Figure 1G). Quantification shows a decrease in Occludin abundance, no significant difference in ZO-1, and a significant increase in BCRP abundance in the hCMEC/D3-BCRP cell line (Figure 1H–J). Additionally, immunofluorescence showed an increase in BCRP expression in the hCMEC/D3-BCRP line relative to parental hCMEC/D3 (Figure 1K). Staining for Occludin and ZO-1 demonstrates preserved junctional expression in hCMEC/D3-BCRP cells (Figure 1K). hCMEC/D3 and hCMEC/D3-BCRP cells showed similar morphology during confluency, with both cell lines forming cobblestone-like endothelial monolayers (Figure 1L). qPCR analysis of additional transporters demonstrated no significant differences in expression of *ABCC1* (MRP1), *ABCC3* (MRP3), or *ABCC4* (MRP4) between hCMEC/D3 and hCMEC/D3-BCRP cells (Figure S2A,C,D). In contrast, *ABCC2* (MRP2) expression was significantly increased in the hCMEC/D3-BCRP cell line, suggesting selective modulation of multidrug resistance-associated transporters (Figure S2B). Taken together, rosuvastatin treatment selectively expanded a population of hCMEC/D3-derived BECs with high BCRP expression.

### 3.2. hCMEC/D3-BCRP Has Increased BCRP Function Compared to hCMEC/D3

**Figure 2:**
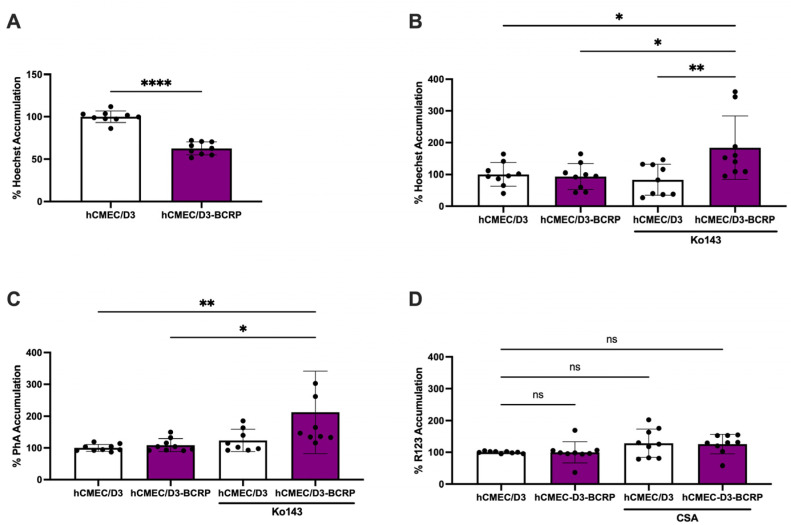
hCMEC/D3-BCRP exhibits increased BCRP function compared with parental hCMEC/D3. (**A**) Substrate accumulation of 10 µM Hoechst is significantly decreased in hCMEC/D3-BCRP cells (purple) compared with hCMEC/D3 cells (white), indicating increased BCRP-mediated efflux. (**B**,**C**) Substrate accumulation assays using 10 µM Hoechst in the presence of BCRP-specific inhibitors (**B**) 1 µM Ko143 or (**C**) 10 µM Pheophorbide A show an increased dynamic range in BCRP inhibition in hCMEC/D3-BCRPs relative to hCMEC/D3s. (**D**) Substrate accumulation of 10 µM R123 in the presence of P-gp inhibitor 10 µM cyclosporine A showed no statistical difference between parent hCMEC/D3s and hCMEC/D3-BCRP cells, indicating no significant change in the dynamic range of P-gp inhibition. All data are expressed as percent accumulation relative to the parental control. Experiments were performed across three independent passages in technical triplicate (*n* = 9). Statistical significance was determined by ordinary one-way ANOVA followed by Dunnett’s multiple comparisons test. Normality tests were performed on all data. The ROUT outlier test (Q = 1%) was applied to datasets, and one outlier with the value of 3668.54717 was removed from (C), *n* = 8. * *p* < 0.05, ** *p* < 0.01, **** *p* < 0.0001, ns, non-significant. Error bars represent SD.

We demonstrated that hCMEC/D3-BCRP overexpresses BCRP, as shown by flow cytometry, qPCR, and immunoblotting (Figure 1). To determine whether the observed increases in BCRP abundance and expression impacted transporter function, we performed substrate accumulation assays using fluorescent BCRP substrates Hoechst 33342 and PhA (Figure 2A–C and Figure S3). First, analysis of Hoechst 33342 accumulation demonstrated that hCMEC/D3-BCRP cells exhibited increased efficiency in excluding the fluorescent substrate relative to parental hCMEC/D3 cells (Figure 2A). Treatment with the BCRP inhibitor Ko143 resulted in increased Hoechst accumulation in hCMEC/D3-BCRP cells (Figure 2B). Similarly, Ko143 treatment increased PhA accumulation in hCMEC/D3-BCRP cells, indicating that the observed effects were not substrate specific (Figure 2C). Next, to assess transporter specificity, we examined P-gp function by measuring the accumulation of the P-gp substrate, Rhodamine 123 (R123). No statistically significant difference in P-gp function was observed between hCMEC/D3 and hCMEC/D3-BCRP cells (Figure 2D). Together, these data demonstrate that hCMEC/D3-BCRP cells exhibit functional BCRP with an expanded dynamic range compared to parental hCMEC/D3 cells, while P-gp function remains unaltered.

## 4. Discussion

Effective drug delivery to the CNS remains a significant challenge due to specialized features of the BBB, particularly high expression and activity of ABC efflux transporters such as BCRP [28]. BCRP serves a major role in maintaining BBB homeostasis through actively limiting the intracellular accumulation of xenobiotic compounds and therapeutic agents [2]. Although this protective function is essential for neurological health, it simultaneously imposes a substantial obstacle for pharmacological intervention in CNS disorders. BCRP was initially identified in drug-resistant cancer cells due to broad substrate specificity, contributing to multidrug resistance, resulting in reduced intracellular clinical substrate concentrations below therapeutic thresholds [6]. Previous research studying multidrug resistance and the role of efflux transporters demonstrated how specific drug compounds, such as Olaparib, temporally alter BCRP and P-gp expression to generate treatment resistance [28]. The parallels between tumor-associated BCRP overexpression and BBB-mediated efflux highlight the relevance of cancer resistance mechanisms for understanding CNS drug exclusion.

Experimental models used to study efflux transport encompass a wide range of systems, including but not limited to rodent, zebrafish, and in vitro approaches. Rodent models offer unique advantages for the study of whole-organism transporter function; however, interspecies differences exist, including variations in BCRP substrate specificity and tissue expression levels. Mice have been shown to exhibit increased baseline expression of Abcg2 and Mdr1a relative to human BBB models [26]. Interspecies human-derived in vitro models allow for directed change in transporter expression and increased ability for high-throughput screenings.

The hCMEC/D3-BCRP cell line is of endothelial origin, and in contrast to overexpression approaches that rely on ectopic expression systems, this model overexpresses BCRP under the control of endogenous promoters. This approach was adapted from a previously published protocol in which hCMEC/D3 cells were exposed to a cytotoxic substrate of P-gp to select for a population of high P-gp expression [18]. Here, hCMEC/D3 cells were treated with the cytotoxic BCRP substrate rosuvastatin at a concentration of 100 µM to generate the hCMEC/D3-BCRP line. After several passages, BCRP expression was validated by flow cytometry, revealing increased BCRP levels relative to the parental cell line. Consistent with these findings, qPCR analysis demonstrated an increased expression of *ABCG2*, the gene encoding BCRP, in hCMEC/D3-BCRP cells. Together, these results demonstrate that prolonged passaging of hCMEC/D3 cells in the presence of rosuvastatin yields a population with elevated BCRP, likely resulting from the selective enrichment of pre-existing subpopulations within the parental hCMEC/D3 line that exhibited higher basal BCRP expression. Rosuvastatin was chosen as the selective agent because it is highly restricted at the BBB by BCRP, with minor contribution from P-gp [29,30]. Additionally, due to its hydrophilicity, passive transport of rosuvastatin across the cell membrane is minimal [31].

In addition to its cytotoxicity at the concentrations used for the development of hCMEC/D3-BCRP cells, rosuvastatin is a hydroxymethylglutaryl-CoA reductase competitive inhibitor used clinically to treat hypercholesterolemia through inhibition of cholesterol synthesis. The mevalonate pathway targeted by rosuvastatin is also responsible for the synthesis of Coenzyme Q10, which is essential for mitochondrial electron transport and energy metabolism [32,33]. As a result of long-term exposure of hCMEC/D3-BCRP cells to rosuvastatin, it is likely that the selection process induced off-target cellular adaptations. Consistent with this possibility, long-term selection for the BCRP overexpressing phenotype was associated with reduced growth rates in the hCMEC/D3-BCRP line compared to the parental line.

Substrate accumulation assays were performed to establish BCRP functionality in hCMEC/D3-BCRP cells relative to parental hCMEC/D3 cells. Following BCRP inhibition, hCMEC/D3-BCRP cells exhibited significantly increased accumulation of two BCRP substrates, Hoechst 33342 and Pheophorbide A, compared to parental hCMEC/D3 cells and untreated hCMEC/D3-BCRP cells. These findings suggest that hCMEC/D3-BCRP cells express higher levels of BCRP and exhibit increased BCRP transport activity. To confirm that P-gp activity was not significantly altered in hCMEC/D3-BCRP cells, P-gp substrate accumulation was measured before and after inhibition with CsA, revealing no significant difference compared to the parental hCMEC/D3 line. This observation highlights the importance of substrate specificity in transporter overexpression models to avoid off-target effects. Endpoint accumulation assays were selected as an initial functional characterization step, consistent with prior BBB transporter model characterization studies [14,18]. Future studies should incorporate concentration-response analyses and bidirectional transport assays to define BCRP substrate affinity, transport capacity, and directional flux in the hCMEC/D3-BCRP cell line. Data will be particularly valuable for translational assessment of CNS drug penetration and for comparison with existing rodent and human BBB models.

Although the hCMEC/D3 cell line is an established model for studying drug transport, it exhibits limitations in tight junction integrity and lacks the cellular complexity of the BBB [13,16,17]. Furthermore, we observed reduced Occludin protein abundance in hCMEC/D3-BCRP cells compared with the parental line. Other BEC efflux overexpression models have reported reduced expression of another junctional protein, VE-cadherin [18]. These effects, along with other potentially uncharacterized adaptations, are important to consider because such changes may limit the broader applicability of this model. Consequently, the addition of neurovascular cues from a co-culture system with other cell types, such as astrocytes and neurons, has the potential to substantially improve the BBB characteristics of this model [34].

Here, we developed a BCRP overexpressing cell line from hCMEC/D3 to mimic BCRP overexpression at the BBB. Increased expression of ABC transporters such as BCRP enhances the utility of hCMEC/D3 cells as a drug transport model and provides a platform with an improved dynamic range for large-scale drug screening. Overall, these findings demonstrate that the hCMEC/D3-BCRP cell line effectively overexpresses functional BCRP without compromising P-gp activity and represents a useful tool for efflux transport studies.

## Data Availability

The original contributions presented in this study are included in the article/Supplementary Materials. Further inquiries can be directed to the corresponding author.

## Supplementary Materials

The following supporting information can be downloaded at: https://www.mdpi.com/article/10.3390/biomedicines14061192/s1, Figure S1. Supporting analysis of BCRP expression in hCMEC/D3-BCRP cells. (A–C) Flow cytometry with gating for unstained, isotype, and BCRP-positive populations. The data demonstrate a greater shift into the gated region for BCRP-positive hCMEC/D3-BCRP cells treated with 100 μM rosuvastatin compared with hCMEC/D3. (D–F) Western blot replicates of Occludin, ZO-1, and BCRP protein expression. Figure S2. ABC transporter expression and stability in hCMEC/D3-BCRP cells. (A,C,D) qPCR analysis of *ABCC1*,* ABCC3*, and* ABCC4* showed no significant difference in the expression of MRP1, MRP3, and MRP4 between hCMEC/D3-BCRP cells and the parental hCMEC/D3 line, indicating that overexpression of BCRP does not alter the expression of these multidrug resistance transporters. (B) A slight increase in *ABCC* (MRP2) expression was observed in the hCMEC/D3-BCRP cells compared to the parental hCMEC/D3 line, suggesting selective modulation of specific ABCC transporters in hCMEC/D3-BCRP cells. (E) qPCR analysis of *ABCG2* expression in hCMEC/D3-BCRP cells across three passages following withdrawal of rosuvastatin from the culture medium demonstrated decreased *ABCG2* transcript levels. Experiments were performed across three independent passages in technical triplicate (*n* = 9). Statistical significance determined by Student’s *t*-test. * *p* < 0.05. Error bars represent SD. Figure S3. Substrate accumulation raw values for data included in Figure 2 comparing hCMEC/D3 and hCMEC/D3 BCRP function. Data presented as RFU/ug.

## Abbreviations

The following abbreviations are used in this manuscript:

BBB	Blood–brain barrier
CNS	Central nervous system
BEC	Brain endothelial cell
BCRP	Breast Cancer Resistance Protein
ABC	ATP-Binding Cassette
P-gp	P-glycoprotein
PhA	Pheophorbide A
CsA	Cyclosporine A
HBSS	Hanks’ Balanced Salt Solution
TEER	Trans-endothelial electrical resistance
R123	Rhodamine 123
PFA	Paraformaldehyde
BCA	Bicinchoninic acid
BSA	Bovine serum albumin
ANOVA	Analysis of variance
cDNA	Complementary DNA
RTC	Rat tail collagen
SD	Standard deviation
