# Supplementary material for: Modeling Blood–Brain Barrier Efflux Transport Using a Breast Cancer Resistance Protein Overexpression Cell Line
Source: Biomedicines. 2026 May 25;14(6):1192. doi: 10.3390/biomedicines14061192 (PMC13295877; doi:10.3390/biomedicines14061192)
Supplement: Supplementary file 1 [file biomedicines-14-01192-s001.zip › Supplement Figure S3.pdf]

	Control		+ Inhibitor	
	**hCMEC/D3**	**hCMEC/D3-BCRP**	**hCMEC/D3**	**hCMEC/D3-BCRP**
R123 Accumulation with CSA	13431.6525	14273.9563	10636.0323	17828.6028
	12904.7058	13030.456	10851.6118	16255.7246
	13068.0505	13320.5258	10250.3698	13900.7842
	5569.56702	59321.9132	7097.39449	36744.2563
	5572.01951	23238.7946	6858.13141	97578.2381
	5213.0534	106732.084	6426.52487	81801.8809
	3078.240301	5649.105839	5290.773942	7515.279609
	3393.969395	5497.082905	5691.455134	8781.869494
	3248.1376	6121.386013	6562.369574	8800.844327
PhA Accumulation with Ko143	691.2604286	768.4771253	715.3200535	970.6303545
	874.7257985	871.0253314	1360.109522	1111.565664
	637.1891024	871.0034897	1025.055658	1221.782872
	10861.98156	38775.13365	434259.1747	171906.3537
	11187.72248	30587.55649	19331.26191	101611.1191
	13462.37916	31255.16874	12111.97889	87769.73262
	0.462329	0.423719	1.224198	1.752975
	0.458361	0.662584	0.98765	1.4459
	0.407444	0.585777	0.985039	1.431422
Hoechst Accumulation with Ko143	474487.9535	87696.60853	470186.9202	195154.3671
	1131867.592	189340.9132	435397.0144	738379.2457
	1940952.21	338152.1081	315712.5757	705424.4981
	77633.27538	59266.76416	74405.15813	61397.64964
	100602.4945	56333.93361	117285.0698	78899.99602
	91404.18746	53480.55043	118420.9052	86012.09166
	259059411.3	251856600.2	867657757.9	344294882.8
	171944683.2	109819633	688966435.4	293156738.8
	119638491.6	80738094.24	225397280	200063815.2
Hoechst Accumulation	77633.27538	59266.76416		
	100602.4945	56333.93361		
	91404.18746	53480.55043		
	1.967874455	1.479054938		
	2.10056555	1.12600292		
	1.95761641	0.896701542		
	1.345403763	0.935515439		
	1.266751542	0.914858706		
	1.283709882	0.919757321		
