# Supplementary figures and images for: Modeling Blood–Brain Barrier Efflux Transport Using a Breast Cancer Resistance Protein Overexpression Cell Line
Source: Biomedicines. 2026 May 25;14(6):1192. doi: 10.3390/biomedicines14061192 (PMC13295877; doi:10.3390/biomedicines14061192)

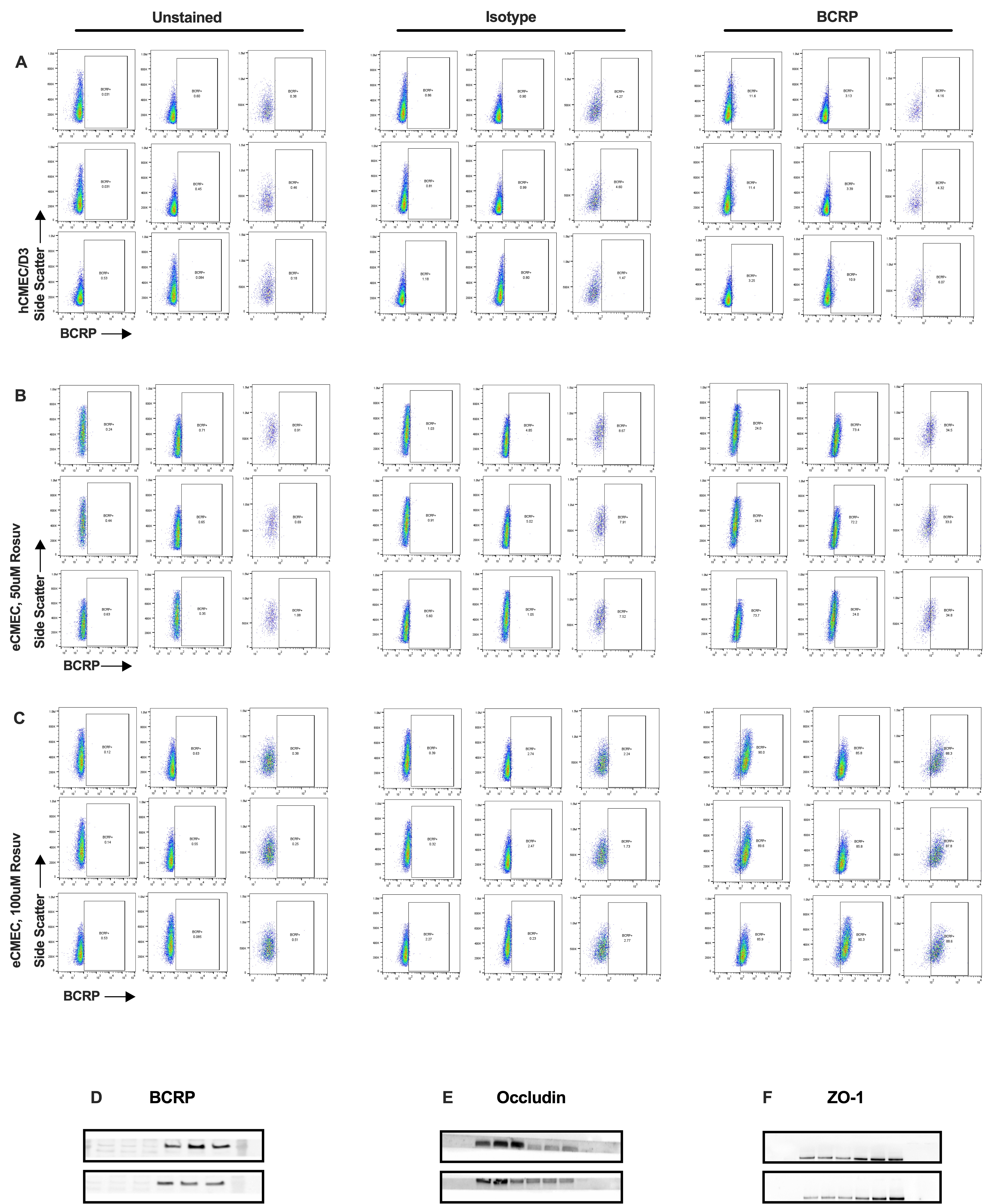

Supplement: Supplementary file 1 [file biomedicines-14-01192-s001.zip › Supplement Figure S1.jpeg]

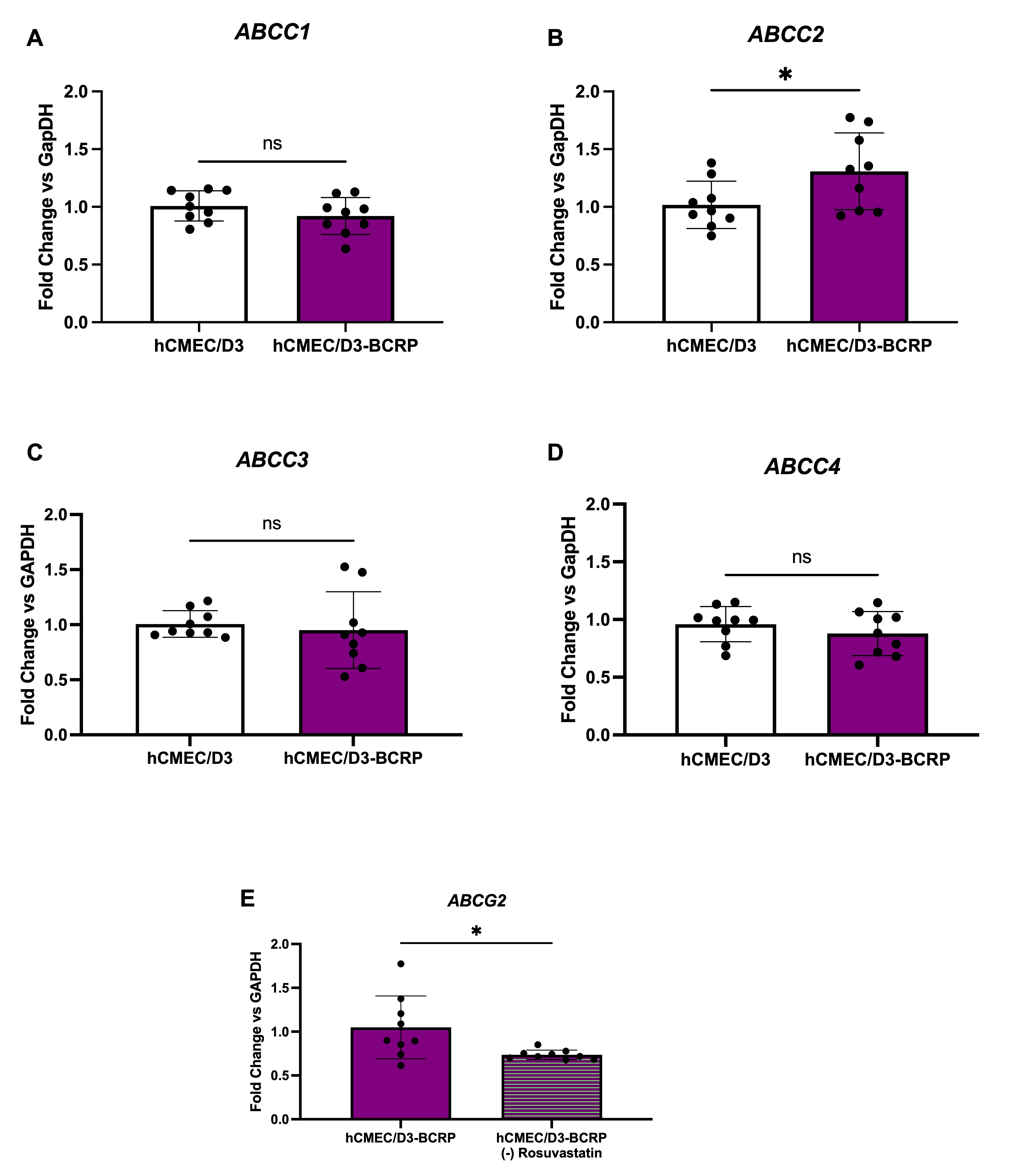

Supplement: Supplementary file 1 [file biomedicines-14-01192-s001.zip › Supplement Figure S2.jpeg]
